# The Multiple Sclerosis (MS) Genetic Risk Factors Indicate both Acquired and Innate Immune Cell Subsets Contribute to MS Pathogenesis and Identify Novel Therapeutic Opportunities

**DOI:** 10.3389/fimmu.2017.00425

**Published:** 2017-04-18

**Authors:** Grant P. Parnell, David R. Booth

**Affiliations:** ^1^Centre for Immunology and Allergy Research, Westmead Institute for Medical Research, University of Sydney, Westmead, NSW, Australia

**Keywords:** multiple sclerosis, genes, vitamin D, Epstein–Barr virus, immune tolerance

## Abstract

Multiple sclerosis (MS) is known to be a partially heritable autoimmune disease. The risk of developing MS increases from typically 1 in 1,000 in the normal population to 1 in 4 or so for identical twins where one twin is affected. Much of this heritability is now explained and is due almost entirely to genes affecting the immune response. The largest and first identified genetic risk factor is an allele from the MHC class II HLA-DRB1 gene, HLA-DRB1*15:01, which increases risk about threefold. The HLA-DRB1 gene is expressed in antigen-presenting cells, and its protein functions in presenting particular types of antigen to CD4 T cells. This discovery supported the development of the first successful immunomodulatory therapies: glatiramer acetate, which mimics the antigen presentation process, and interferon beta, which targets CD4 T cell activation. Over 200 genetic risk variants, all single nucleotide polymorphisms (SNPs), have now been described. The SNPs are located within, or close to, genes expressed predominantly in acquired and innate immune cell subsets, indicating that both contribute to MS pathogenesis. The risk alleles indicate variation in the regulation of gene expression, rather than protein variation, underpins genetic susceptibility. In this review, we discuss how the expression and function of the risk genes, as well as the effect on these of the risk SNPs, indicate specific acquired immune cell processes that are the target of current successful therapies, and also point to novel therapeutic approaches.

## Introduction

Multiple sclerosis (MS) is an autoinflammatory disease in which the oligodendrocytes are destroyed and neuronal function is progressively lost (1). Risk is greatly increased with increasing relatedness to someone who has MS. The genetic basis for this increase in risk has been largely determined by genome-wide association studies (GWAS), which indicates that common variation in the regulatory regions of immune genes largely drives variation in susceptibility to MS (2). Over 200 genes have now been identified (3) and, of these, 110 non-MHC genetic loci have been detailed (2) and 13 MHC loci identified (4).

The heritability of a disease is the proportion of total variance in disease risk that is explained by genetic variance (5). A recent meta-analysis of twins concluded that genetic variation may be responsible for about half of the individual differences in susceptibility to MS (6), further supported by a large national study (7). This is similar for many common autoimmune diseases (8). The c.200 risk genes identified from GWAS, using genotyping from more than 100,000 cases and controls, is estimated to account for toward half of MS heritability (3).

These MS risk variants are expressed in a wide range of immune cell types (9), indicating that multiple immune cell types contribute to the immune dysregulation that alters susceptibility to MS. This is consistent with successful MS therapies targeting highly different immune cell types: a monoclonal antibody to CD20 is B cell specific (10); to CD25 is T/NK/MP cell specific (11); and others are pan immune [CD52 (12); CD49d (13)]. Of these therapeutic targets, both CD25 and the ligand for CD49d (VCAM1) are MS risk genes. This suggests that many therapeutics could be employed targeting the other 200+ MS risk variants, and that novel therapies targeting specific immune cell types and states identified by the risk genes should be possible. Other therapies alter migration to the central nervous system (CNS) by retaining multiple immune cell types in secondary lymphoid organs [S1PR agonists; (14)], or by altering immune cell physiological state [Tekfidera; (15); Teriflunomide; (16)]. All therapies fail in a proportion of patients, with resulting CNS damage and significant economic cost (17).

Environment also contributes to MS susceptibility. These include latitude of childhood, age at Epstein–Barr virus (EBV) infection, salt, and smoking (18). Each of the environmental risk factors can be manipulated or their effects modified through understanding how they affect immune response. Similarly, although an individual’s genetic risk factors cannot yet be altered, their consequences on immune response can be manipulated through an understanding of how they affect MS risk, including how they interact with environmental risk factors.

In this review, we discuss how the known MS genetic risk factors may affect the acquired immune response, and how this points to novel therapeutic strategies. Unlike Mendelian diseases, single genetic effects are small in MS. However, they point to processes and cell subsets necessary for MS pathogenesis, and as mentioned above, targeting single genes tagged by their albeit small risk factors has proved highly effective in reducing disease. Defining the gene, and the cell subset and state it in turn tags, should be beneficial in improving therapy. This is particularly likely given the recent discoveries that many key immune cell populations are highly heritable (19–21).

Our approach in this review is to consider the immune cell subsets in which the MS risk genes are most highly expressed, as these are the most likely to underpin the risk genes’ contribution to pathogenesis. The risk genes typically control differentiation and state of the immune cells and act through their function on particular cellular processes in these cells. These cellular genetic effects are modulated through the effect of the risk single nucleotide polymorphisms (SNPs). The context and consequences in which these SNPs exert their effects are often difficult to determine, especially for pleiotropic molecules. The SNP effect may be within the major immune cell type in which the risk gene functions, or be due to the balance of many risk gene effects on the immune response, and may be highly context specific, such as on infection at a particular tissue location and time.

A consilient approach, where the more genetic factors that point to a particular process as being pathogenic the more likely it is to be true, can be facilitated by considering how these genetic risk factors might function to mediate environmental risk. Consequently, in this review, we have focused on how the effects of the genes on the acquired immune cell state, function, and differentiation might be shared with the effects of environmental risk factors; and how this might contribute to the development of novel therapeutic approaches.

## The First Risk Gene, HLA-DRB1*15:01

The first MS risk gene variant, HLA-DRB1*15:01, increases risk by threefold (22). The others confer an increased risk of less than 1.2-fold. Although the increased risk of the gene variant does not necessarily indicate the relative importance of the risk gene in pathogenesis, it does indicate the relative effect of the genetic variant haplotype. The HLA-DRB1 variant 15:01 therefore, affects pathogenesis more than the other known risk variants, but the relative importance of other risk genes to pathogenesis is unknown. HLA-DRB1 has limited and well-known roles: it is expressed in antigen-presenting cells, and it presents peptides to CD4 T cells in the process of their regulation, both activation and inactivation. Consequently, we can conclude CD4 T cells are important in pathogenesis, and the peptide presented by DRB1*15:01, and/or the regulation of this variant, are highly important to pathogenesis. From protein prediction studies, these peptides are hydrophobic. Many myelin sheath proteins have highly hydrophobic peptides. Unlike other DRB1 alleles, the structure of the 15:01-binding groove has been shown to present both myelin and EBV peptides to T cells (23). Molecular mimicry (where non-myelin peptides select the same T cell clones) and epitope spreading (where related T cell clones are selected by peptides similar to the one initiating an immune response) could contribute to T cell activation through DRB1*15:01. A pathogen such as EBV could drive such immune activation. Tschochner et al. (24) have recently demonstrated a number of potential cross-reactive targets of major myelin antigens and EBV proteins. Antigen presentation occurs in the thymus, where the CD4 T cell repertoire is restricted, and where regulatory T cells are selected. It also occurs in the secondary lymphoid organs, notably, the draining cervical lymph nodes, which present antigen from the CNS to naïve T cells, resulting in their activation or inactivation (tolerance, cell death, and regulatory T cells). CD4 T cells will also recognize antigen in the CNS, including that presented by antigen presenting cells (APCs) there. Finally, the B cell arm of the immune response will be activated by CD4 Th2 cells in germinal centers of the secondary lymphoid organs, elsewhere, and in the tissues. B cells can also act as antigen-presenting cells. The HLA-DRB1*15:01 allele is associated with phenotypic features of the disease including female sex and presence of cerebrospinal fluid-restricted oligoclonal bands, and the HLA genetic burden has now been associated with several MRI traits (25). Other HLA *DRB1* loci are also independently associated with MS risk (4).

**Figure 1:**
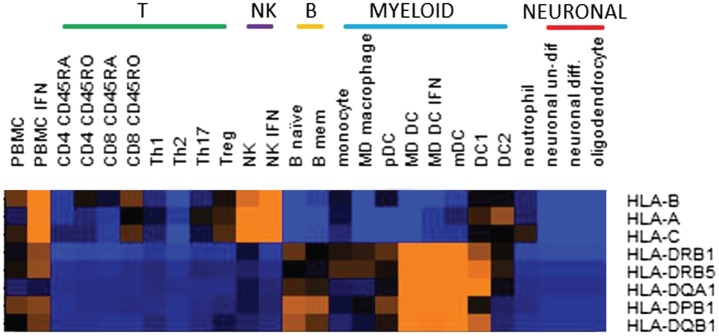
**Relative expression in cell subsets of the multiple sclerosis risk genes located in the major histocompatibility complex region**. Expression was by RNASeq and color on heatmap indicates relative expression level: orange is high, blue is low. MHC risk genes are identified in Ref. (4). Cell subsets were *ex vivo* or *in vitro* generated as previously described (26).

Several MHC class I alleles have also been identified, all with protective effects (4). These present antigen to CD8 T cells, or interact with natural killer (NK) cells. CD8 T cells responding to antigens presented by these protective alleles may be more effectively activated to kill EBV infected cells. Or, these alleles could facilitate superior regulation by NK cells of target cells, such as infected cells, or autoreactive T cells. These processes might be augmented by therapies or vaccines. It is interesting that although HLA A/B/C genes might be expected to be expressed in a wide range of cell types, of immune cells *ex vivo*, we found that their expression was highest in NK cells (Figure 1). Higher expression of these risk alleles in NK cells might indicate their importance in regulating the NK cells response, at least in the homeostatic conditions tested, and suggests an interesting area for further investigation.

Risk gene effects, both MHC and non-MHC, appear to be additive, with little evidence for interactive effects (2, 4). An exception is two interactions between pairs of class II alleles: HLA-DQA1*01:01–HLA-DRB1*15:01 and HLA-DQB1*03:01–HLA-DQB1*03:02 (4). These authors found no evidence for interactions between classical HLA alleles and non-HLA risk-associated variants. Genetic load, including just the MHC arm (27) can predict altered MS susceptibility and phenotypes, but have little diagnostic utility, since even the few people with unusually high MS genetic loads are unlikely to develop MS.

## Risk Gene Immune Cell Phenotypes

The possibility that MS risk genes define immune cell population differences that contribute to pathogenesis has been supported by the recent finding that many immune cell populations are highly heritable (19–21). Differences in differentiation of immune cells between individuals are controlled by genetic variation, e.g., of transcription factors, cytokine receptors, and signaling molecules. MS risk genes such as TYK2 (28, 29), IL2Ra (30), EOMES (31), NFKB1 (32), and ZMIZ1 (33) are associated with immune cell population differences in MS. Increased understanding of immune dysregulation in MS, the nature of control of this dysregulation, and identification of points for therapeutic intervention will come from further investigation of how risk genes and alleles affect heritable immune cell populations. For example, Hartmann et al. found a significantly increased propensity of T_H_ cells from individuals carrying *IL2RA* risk alleles to secrete GM-CSF, and that such cells were more abundant in MS (30). GM-CSF neutralization trials are also currently ongoing in MS patients (NCT01517282). GM-CSF B cells (34) may yet prove to be driven by MS risk factors. These populations are therapeutic targets, and agents exist that can modulate each of them: novel agents may prove to be more specific to the MS-promoting aspect of these populations. The latter needs to be determined by further study.

Brodin et al. (20) also demonstrated that the difference in response of immune cell subsets to the cytokines IL7 and IL2 could be highly heritable. These genes and/or their receptors are MS genetic risk factors (35). Genes such as these defining heritable immune cell subsets and responses are good candidates for drug targets and biomarkers. It is notable that the percentage of lymphocytes in peripheral blood-expressing CD4 is highly heritable and predicts response to fingolimod (36).

## Cell Subset and State

**Figure 2:**
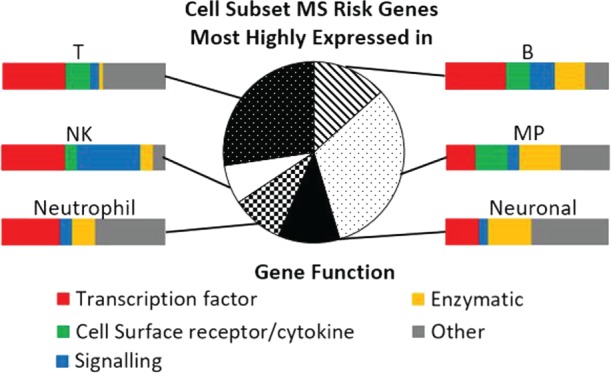
**Cell subset expression and function of multiple sclerosis (MS) risk genes**. Pie chart indicates the proportion of MS risk genes that are most highly expressed in each of the cell subsets, as measured by RNAseq. Details of cell isolation and RNAseq workflow are described in Ref. (26). Bar charts illustrate the function of the genes most highly expressed in each of the cell subsets. MP, mononuclear phagocytic; NK, natural killer. Gene function was categorized using the Entrez gene summary and gene ontology annotations for each gene.

Antigen-presenting cells and CD4 T cells are implicated by the HLA-DRB1 genetic association; CD8 and NK cells by the MHC class I associations. These and other cell types are also implicated by the non-HLA MS risk genes (Figure 2) (4, 9, 35). The general immune cell pattern of cells expressing risk factor genes indicate that a wide range of subsets and contexts are likely to contribute to disease development. For example, pathogenic studies have implicated autoreactive CD4, CD8, and B cells; and the cells that regulate them including regulatory T (many types), regulatory B (many types), the cells that regulate all of these [collectively the mononuclear phagocytic (MP) cells]; as well as NK cells (37). Each individual may have dysregulation in a particular combination of these, explaining at least some of the variation in therapeutic response. The risk genes dominantly expressed in these major subsets include transcription factors, chemokines, receptors, and intracellular enzymes and signaling factors (Figure 2), which would be expected to not only control the proportion, differentiation, and state of these subsets (Table 1) but also provide therapeutic targets for manipulating them. By identifying how these risk variants affect gene function, off-the-shelf therapeutic agents already available for the risk genes could be potentially repurposed to MS, especially for cell surface receptors.

**Table 1:** **Gene ontology (GO) pathways overrepresented in the lists of multiple sclerosis risk genes most highly expressed in T, B, mononuclear phagocytic (MP), and natural killer (NK) cells, respectively**.

Cell type	GO pathways overrepresented (*p*-value)
T	Differentiation (1E-18), activation (1E-17), cell adhesion (1E-16)
B	Chemokine secretion (1E-9), activation (1E-8), migration (1E-8)
MP	Activation (1E-8), adhesion (1E-7), differentiation (1E-6)
NK	Activation (1E-8), STAT cascade (1E-7)

*p-Values were calculated using GeneGo MetaCore*.

As well as those immune populations described in Section “Risk Gene Immune Cell Phenotypes,” some progress has been made in determining the effects of particular SNPs and genes. The IL2R risk variants control splicing and affect the proportion of GM-CSF-producing T helper cells (30). The IL7R risk variant affects splicing, affects T cell repopulation after lymphopenia in transplants (38) and HIV (39), and could also affect T regulatory cell proportion and function (40). Several other genes support dysregulation of Tregs as pathogenic, notably the transcription factors BACH2, IKZF1, and IKZF3.

Immune effects of risk genes are likely more easy to identify than the specific effects of SNPS, but the latter has indicated why therapies are useful in some autoimmune diseases, but exacerbate them in others. For example, the MS CD40 risk variant of SNP rs6074022 is protective for rheumatoid arthritis, and monoclonal antibodies to CD40 are effective for the latter, but exacerbate disease in the former (41). Similarly, opposite associations of the TNFRSF1A allele rs1800693 correspond to opposite outcomes of anti-TNF in treatment for MS and other autoimmune diseases (42).

Although it is been widely considered that the genetic associations with MS provide a roadmap to understand pathogenesis and devise new therapeutic strategies, most of that map has not been exploited for the investigation of how the genes and their variants affect pathogenesis.

## Interaction with Environmental Risk Factors: Therapeutic Implications

Multiple sclerosis risk is greatly reduced in low latitudes, and this has been attributed to the effects of ultraviolet light, including the production of vitamin D, leading to clinical applications (43). An immediate benefit of the identification of the first 110 MS risk genes was the compelling evidence that vitamin D regulation contributes to MS susceptibility: the risk gene CYP24A1 inactivates vitamin D, and the risk variant increases this inactivation in dendritic cells (26). The vitamin D-activating gene CYP27B1 is also implicated, and its risk variant is less active in tolerizing dendritic cells. Other vitamin D-regulated genes in MP cells have been identified (44), and there is an overrepresentation of genes associated with MS and other latitude-dependent autoimmune diseases. These data have supported the now widespread use of vitamin D supplementation in clinical management of MS. By indicating the immune cells mediating the vitamin D protection, and the genes regulated by vitamin D in these immune cells, this genetic finding also provides a tool to dissect out the molecular architecture of vitamin D control of tolerance. Vitamin D supplementation can be implemented in a variety of ways. Optimal and improved methods to stimulate tolerance, and to monitor vitamin D sufficiency by immune cell readout, may result from further investigations of the regulation of the vitamin D pathway.

Common disease-associated variants affect expression of pathogen-sensing genes in dendritic cells, highlighting the importance of infection on driving functional variation that also affects disease (45). There is a strong evidence that the EBV is necessary for development of MS (46), with compelling evidence for causation (47). However, most of those infected never develop MS. Some MS risk genes would be expected to indicate differences in the immune response to EBV that contribute to MS risk. The paradox of the high expression of the T cell-activating gene CD40 being protective in MS may be explained by its role in EBV proliferation in B cells (9). The risk gene TRAF3 also functions on this signaling pathway. The risk genes ZMIZ1 (33) and EOMES (48) are associated with levels of antibodies to EBV. Given that the recent dramatic success of B cell therapies may be due to their effect on EBV-infected B cells (49), identifying host genetic factors affecting immune control of EBV and MS risk may prove particularly promising areas of investigation.

Further, environmental risk factors (EBV, smoking, and obesity) increase the risk of MS with combinations of the MHC risk factors (HLA-A*2 protective, DRB1*15:01 risk) greatly beyond the additive effects of these genetic risk factors: additive risk 5.0, interactive risk 14 (50). This suggests that there is an increased risk of MS due to the antigen-presentation pathways when environmental factors increase the effects of an immune response and highlight the value of interventions based on reducing these environmental risk factors.

## Conclusion and Future Work

Although much of the heritability of MS has been discovered, the current findings have not yet been sufficiently exploited. Some progress has been made in the identification of pathogenically significant changes to immune cell types, state, and differentiation; and interaction with UV light and vitamin D. Design of new therapies for risk gene-guided immunomodulation, repurposing of existing drugs, better use of vitamin D analogs and methods of use, and guided use of current drugs should be facilitated by knowledge of the pathogenic processes tagged by the risk factors.

## Author Contributions

DB drafted the manuscript, GP the figures, and both edited the final version.

## Conflict of Interest Statement

The authors declare that the research was conducted in the absence of any commercial or financial relationships that could be construed as a potential conflict of interest.
